# The additive value of preprocedural cardiac computed tomography angiography (CCTA) planning in percutaneous left atrial appendage occlusion: a systematic review and meta-analysis

**DOI:** 10.1007/s10840-026-02273-2

**Published:** 2026-03-28

**Authors:** Elsayed Balbaa, Mohamed Saad Rakab, Ahmed Farid Gadelmawla, Ahmed Mazen Amin, Abdelrahman M. Tawfik, Abdelrahman Elgendy, Alaa R. AL-Ihribat, Mustafa Turkmani, Obieda Altobaishat, Mohamed S. Elgendy, Abdalhakim Shubietah, Mohamed Abuelazm

**Affiliations:** 1 Faculty of Medicine, Alexandria University, Alexandria, Egypt; 2 Faculty of Medicine, Mansoura University, Mansoura, Egypt; 3 Faculty of Medicine, Menoufia University, Menoufia, Egypt; 4 Faculty of Medicine, Palestine Polytechnic University, Hebron, Palestine; 5 Faculty of Medicine, Michigan State University, East Lansing, MI USA; 6 Division of Pulmonary and Critical Care, University of Toledo, Toledo, OH USA; 7 Faculty of Medicine, Jordan University of Science and Technology, Irbid, Jordan; 8 Faculty of Medicine, Tanta University, Tanta, Egypt; 9 Department of Medicine, Advocate Illinois Masonic Medical Center, Chicago, IL USA

**Keywords:** Cardiac imaging, Cardiac computed tomography, CCTA, Left atrial appendage closure, LAAC, Atrial fibrillation

## Abstract

**Background:**

Cardiac computed tomography angiography (CCTA) is increasingly used to guide left atrial appendage occlusion (LAAO) by providing a detailed anatomical assessment. However, its incremental value over conventional planning methods, such as transesophageal echocardiography or fluoroscopy, remains uncertain. This study evaluated the additive value of CCTA-guided planning in percutaneous LAAO.

**Methods:**

A systematic review and meta-analysis conducted using PubMed, Embase, CENTRAL, Scopus, and Web of Science, up to September 2024 (updated in July 2025). Dichotomous outcomes were pooled as risk ratios (RR), while continuous outcomes were pooled as mean differences (MD) along the corresponding 95% confidence intervals (CI). PROSPERO ID: CRD42024600363.

**Results:**

Six studies comprising 978 patients with follow-up periods of up to 90 days were included. The CCTA-guided group had significant higher device implantation success [RR: 1.06; 95% CI (1.01, 1.11), *P* = 0.028], a lower device resizing [RR: 0.42; 95% CI (0.26, 0.66), *P* = 0.0002], and a lower procedure time [MD: -13.29 min; 95% CI (-23.72, -2.86), *P* = 0.01]. There was no significant difference between the two groups in device-related embolization [*P* = 0.38], peri-device leakage [*P* = 0.16], radiation dose [*P* = 0.84], and contrast utilization [*P* = 0.96].

**Conclusion:**

Adding CCTA to preoperative planning for LAAO as a complementary modality can increase implantation success rate, improve device selection accuracy, and shorten procedure time without increasing adverse events or contrast use with substantial heterogeneity and divergent subgroups. However, limited follow-up restricts long-term safety conclusions. Further large-scale, long-term randomized trials are needed to validate these findings.

**Graphical Abstract:**

**Figure:**
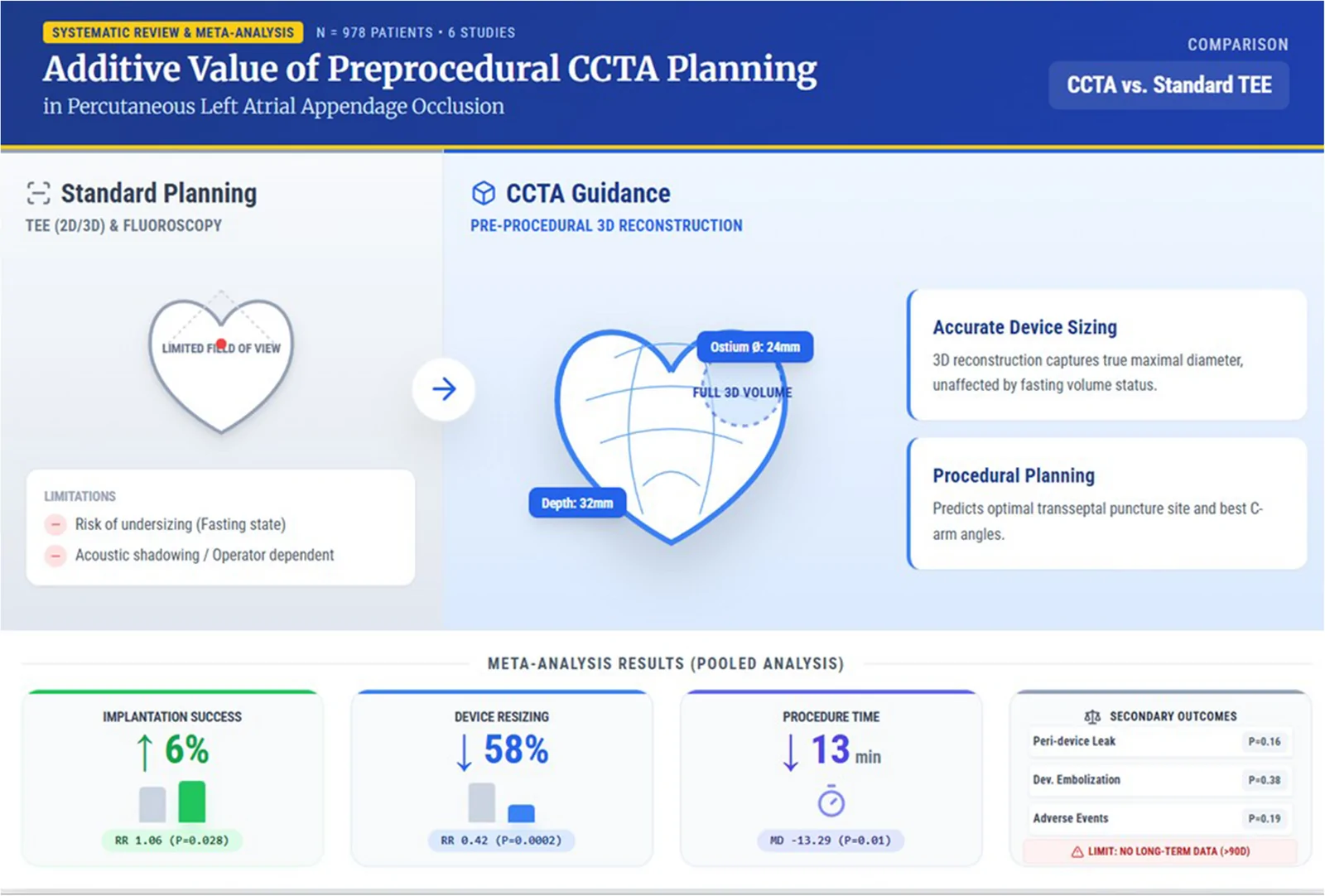

**Supplementary Information:**

The online version contains supplementary material available at https://doi.org/10.1007/s10840-026-02273-2.

## Introduction

Atrial fibrillation (AF) represents a significant burden. In 2019, the number of affected individuals exceeded 59 million. Moreover, atrial flutter or fibrillation will affect around thirty-three million individuals and result in around 2.5 million deaths between 2030 and 2034 [1]. AF is a significant cause of ischemic stroke, which often results in death or disability [2]. AF is increasingly understood as a systemic disorder driven not only by electrophysiologic abnormalities but also by extracardiac factors such as inflammation, metabolic dysregulation, and gut–heart axis alterations [3, 4]. Current guidelines state that rhythm control techniques, such as catheter ablation, are essential for managing atrial fibrillation (AF), particularly when the condition is symptomatic or persistent. Among available ablation procedures, both cryoballoon and radiofrequency ablation have demonstrated equivalent efficacy and safety, with cryoballoon ablation offering the advantage of a shorter procedure time [5, 6]. Apart from rhythm control techniques, current guidelines recommend the use of direct oral anticoagulants for nearly all AF patients, with growing evidence about the potential use of other drugs, including Factor XI/XIa inhibitors [7, 8]. In patients with AF, most of the thrombi originate in the left atrial appendage (LAA) [9]. Therefore, the European Society of Cardiology recommended LAA occlusion for stroke prevention in patients with AF and contraindications to anticoagulants [10]. The most used devices in LAA occlusion are the Amulet, Watchman, and Amplatzer cardiac plug. 

The LAA is a finger-like extension from the left atrium and is commonly present at the anterolateral sides of the left atrium. However, the anatomy of the LAA varies from one individual to another. Therefore, adequate imaging and pre-procedure assessment of the LAA are essential [11]. The gold standard for perioperative evaluation of LAA is transesophageal echocardiography (TEE) [12]. However, TEE is an invasive procedure [11]. Furthermore, it has been shown that using TEE imaging, more than half of the patients initially received an incorrect device [13]. Therefore, there is a need for an accurate imaging modality.

Cardiac computed tomography angiography (CCTA) is a non-invasive imaging modality that provides high-resolution 3-dimensional images. Thus, it could help exclude LAA thrombosis, evaluate the anatomy of LAA, and determine the accurate device size [14]. Recently, it has been shown that CCTA in patients undergoing LAA occlusion (LAAO) could improve the device’s success rate [13]. However, the effect of using CCTA in the pre-procedural planning of LAAO is still unclear; only a few studies have investigated the efficacy and safety of this approach [15–18]. Therefore, we conducted this systematic review and meta-analysis to investigate the effect of adding CCTA to the pre-procedural assessment of patients undergoing LAAO.

## Methodology

### Protocol registration

Our review procedure was registered and published in PROSPERO under the ID CRD42024600363. We conducted a systematic review and meta-analysis, in accordance with the Preferred Reporting Items for Systematic Reviews and Meta-Analyses (PRISMA) statement [19] and the Cochrane Handbook [20].

### Data sources & search strategy

The studies included in our review were identified through a systematic search of the Web of Science, SCOPUS, PubMed (MEDLINE), Cochrane Central Register of Controlled Trials (CENTRAL), and clinicaltrials.gov from their inception until September 2024 (updated in July 2025). Neither search filters nor language restrictions were used. The search strategy is demonstrated in Table S1.

### Eligibility criteria

We included studies only with the following PICO criteria:

All studies with human participants, regardless of their gender or demographic characteristics, were included. Studies utilizing CCTA guidance compared to non-CCTA guidance (defined as patients receiving standard-of-care planning and guidance, primarily utilizing TEE (2D or 3D, and pre-procedural or intra-procedural) and/or fluoroscopy) in patients undergoing LAAO were included. Finally, outcomes related to implantation success, need for device resizing, compression, peri-device leak, total procedural time, radiation dose, contrast utilization, or intraoperative per-case angiographic positions were accepted.

The exclusion criteria for the papers were as follows: book chapters; non-human and in vitro experiments; studies with overlapping or duplicate datasets; reviews; comments; letters to the editor; guidelines; and studies with a single arm. Any publications lacking primary data and/or explicit method descriptions were also excluded.

### Study selection

Search results from all databases were imported into Covidence.org, and duplicates were automatically removed. Four authors independently screened the remaining records (A.M.T., A.E., A.M.S., and A.R.A.), and a fifth author (M.S.R.) resolved any conflicts. The screening was conducted in two phases: (i) screening the title and abstract to determine the study’s relevance to this meta-analysis and to remove ineligible articles. (ii) full-text screening according to the inclusion criteria for the final qualitative and quantitative analysis eligibility.

### Data extraction

Data were collected independently by four review authors (A.M.T., A.E., A.M.S., and A.R.A.) and extracted into a uniform data extraction Excel sheet. Data extraction included (i) a summary of the study features, including but not limited to (study design, country, number of centers, total number of participants, contrast and device used, primary outcome, and follow-up duration). (ii) baseline characteristics of the population upon trial, including (the number of patients in each group, age, gender, BMI, heart failure, ejection fraction, and CHA₂DS₂-VASc score). (iii) post-intervention outcomes, including (implantation success, need for device resizing, compression, peri-device leak, total procedural time, radiation dose, contrast utilization, or intraoperative per-case angiographic positions). All data extraction and database typing were reviewed by a fifth reviewer (M.S.H) before the final analysis. Any disagreement was resolved by consensus. We converted medians and interquartile ranges (IQRs) into means and standard deviations using the Meta-Analysis Accelerator tool, a reliable platform for statistical data conversion that uses standard formulas [21].

### Risk of bias assessment

Four reviewers (A.M.T., A.E., A.M.S., and A.R.A.) independently assessed the quality of the included studies. We used the Cochrane ROB2 tool for RCTs [22]. The evaluated domains included bias from the randomization process, bias from deviations from the intended intervention, bias from missing outcome data, bias in outcome measurement, and bias arising from selective outcome reporting. The ROBINS-I tool was used for observational studies [23].

### Statistical analysis

The study employed R version 4.3, utilizing the “meta”, “metafor”, and “deter” packages for statistical analysis. The analysis combined results from multiple studies, using either risk ratios (for dichotomous outcomes) and mean differences (MD) or standardized mean differences (SMD) (for continuous outcomes), all with 95% confidence intervals. A random-effects model using the restricted maximum likelihood (REML) method was applied regardless of I^2^ to account for anticipated between-study heterogeneity. Heterogeneity was interpreted according to the Cochrane Handbook (chapter nine) [20], with I2 values of 0–40% indicating low heterogeneity, 30–60% moderate heterogeneity, 50–90% substantial heterogeneity, and 75–100% considerable heterogeneity. A Chi-square test p-value below 0.1 was considered statistically significant for heterogeneity. We also performed a sensitivity analysis using the leave-one-out method, an approach that involves sequentially omitting one study at a time, to investigate the source of heterogeneity and assess the overall robustness of estimates [20]. A prespecified subgroup analysis according to study design was conducted whenever possible. A subgroup analysis by device type was not feasible because most included studies used only the WATCHMAN device, and there were insufficient data on other devices. Finally, a test for publication bias was not possible as we included fewer than 10 studies [24].

## Results

### Search results and study selection

**Fig. 1:**
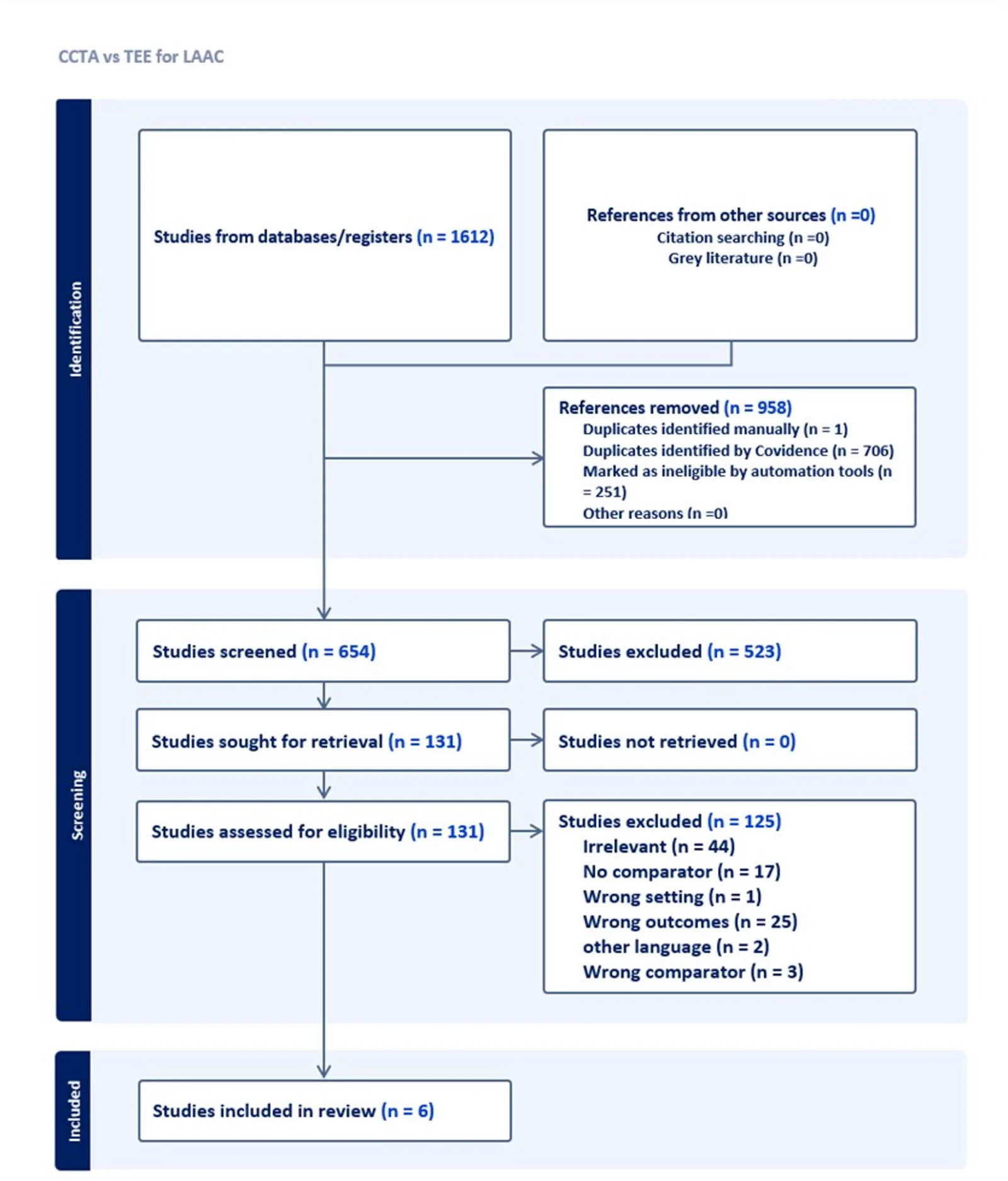
PRISMA flowchart of the included studies

Our search strategy retrieved 1,612 studies. After title and abstract screening and removing duplicates, 1,481 articles were eliminated, and 131 full-text articles were further evaluated for eligibility. Six studies [15–18, 25] met our criteria following full-text screening and were included in our meta-analysis. The PRISMA flowchart is shown in Fig. 1.

### Characteristics of included studies

Six studies [15–18, 25, 26] comprising 978 patients were included in our comprehensive systematic review and meta-analysis. The included studies assessed LAAC using the WATCHMAN device, with one study adding the Amplatzer Amulet to its design and another using the Amplatzer Amulet device exclusively. Three studies were retrospective (Meng et al., 2024; So et al., 2021; and Soh et al., 2024), while the others were randomized clinical trials (Eng et al., 2017; Galea et al., 2023; and Wang et al., 2023). Sample sizes ranged from 24 (Eng et al., 2017) to 485 (So et al., 2021). Most studies were conducted in China, except for Eng et al. (USA), Galea et al. (Italy, Switzerland, France), and Soh et al. 2024 (Ireland). Follow-up durations ranged from 45 to 90 days, though Meng et al. (2024) and Soh et al. (2024) did not report follow-up. For pre-procedural evaluation and device size, the included studies used a variety of imaging procedures. Pre-procedural TEE was utilized in most studies (So et al., Wang et al., Galea et al., Soh et al.) to screen for and rule out spontaneous echo contrast or LAA thrombus. Cardiac computed tomography angiography (CCTA) was then or concurrently performed and used exclusively for a thorough anatomical evaluation of the LAA and device size, whereas in Galea et al., the device size was selected using preprocedural CCTA in addition to fluoroscopy and echocardiography. One study (Eng et al.) did not use pre-procedural TEE to exclude thrombus; instead, it used pre-procedural CT for anatomical assessment. For guidance and post-procedural evaluation, all studies used intra-procedural TEE; some also used fluoroscopy.

### Risk of bias assessment

According to the ROB 2 tool, Wang et al. and Galea et al.[25] were rated as having low risk of bias, whereas Eng et al.[15] raised concerns about deviations from intended interventions, as the imaging cardiologists were not technically blinded (Figure S1). The ROBINS-I assessment tool for observational studies was also used, showing a moderate risk of bias in Meng et al.[16], a high risk in Soh et al.[26], and a serious risk in So et al.[18] (Table S2).

### Primary outcomes

#### Implantation success

**Fig. 2:**
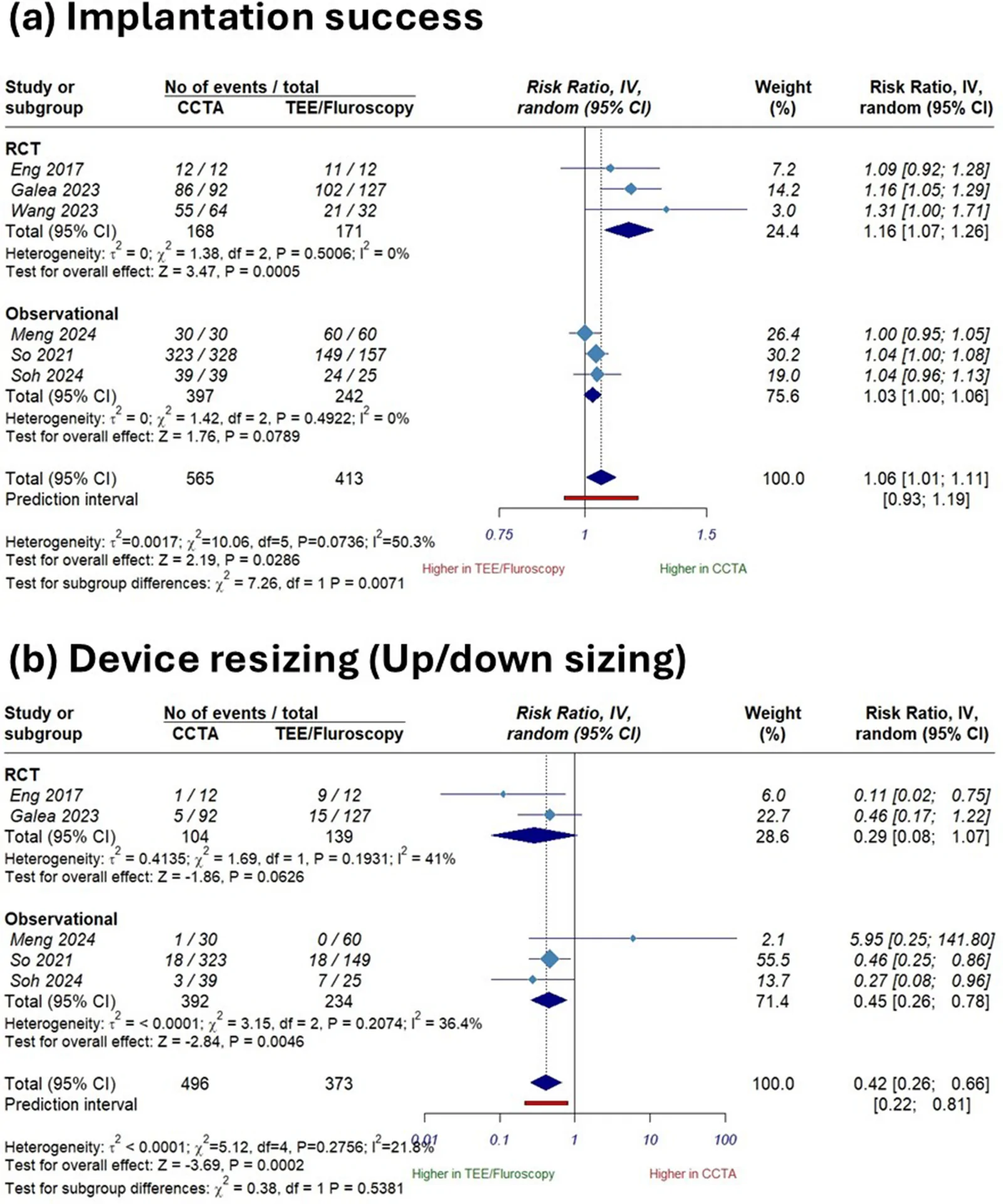
Forest plot of: Implantation success (**a**) and Need for device resizing (**b**)

CCTA was associated with a statistically significantly higher rate of implantation success compared to TEE/fluoroscopy (RR: 1.06, 95% CI [1.01, 1.11], *P* = 0.028; Fig. 2a). Heterogeneity was moderate (I² = 50.3%, *P* = 0.07). Sensitivity analysis suggested that omitting Galea et al. 2023 [25] reduced heterogeneity (I² = 19%), with no effect on statistical significance (RR: 1.03, 95% CI [1.001,1.06]; Figure S2). By study design, RCTs were associated with a significant increase in implantation success (RR 1.19, 95% CI [1.08–1.31]), while observational studies showed a non-significant increase (RR 1.03, 95% CI [1.00–1.07]). Subgroup differences were significant (*P* = 0.0007).

#### Need for device resizing

CCTA was associated with a lower rate of device resizing compared to TEE/fluoroscopy (RR: 0.42, 95% CI [0.26, 0.66], *P* = 0.0002; Fig. 2b). Low heterogeneity was observed across studies (I² = 21.8%, *P* = 0.27). The sensitivity analysis using the leave-one-out method showed robustness of the overall results, as no single study omission affected the direction or significance of the results (Figure S3). Subgroup analysis by study design showed that the significant results were maintained only in the observational design subgroup, not in the RCT subgroup.

### Secondary outcomes

#### Compression

**Fig. 3:**
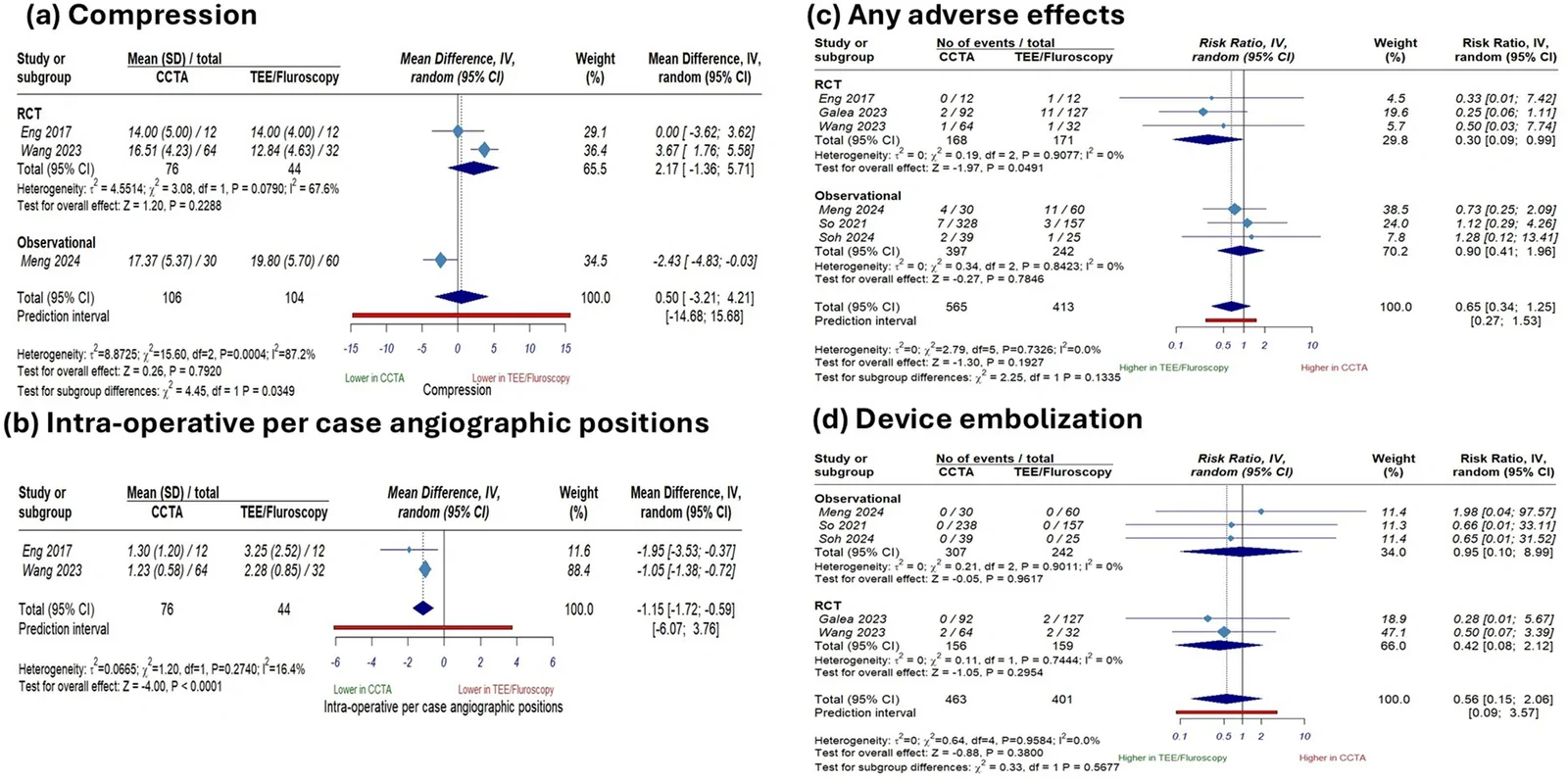
Forest plot of: Compression (**a**), Intra-operative per case angiographic positions (**b**), Any adverse events (**c**), and Device embolization (**d**)

There was no significant difference between CCTA-derived modeling and TEE/fluoroscopy regarding compression (MD: 0.50, 95% CI [-3.21, 4.21], *P* = 0.79; Fig. 3a). The analysis showed high heterogeneity (I² = 87.2%, *P* = 0.00004). Leave-one-out sensitivity analysis revealed that omitting Wang 2023[17] markedly reduced heterogeneity (I² = 16.7%; Figure S4), with no effect on significance. By study design, RCTs showed consistent results with the overall analysis (MD 2.17, 95% CI [-1.36, 5.71]), while observational studies showed a significant reduction (MD -2.43, 95% CI [-4.83, -0.03]). Subgroup differences were significant (*P* = 0.03).

#### Intraoperative per-case angiographic positions

CCTA was significantly associated with a reduced number of intraoperative angiographic positions per case compared to TEE/fluoroscopy (MD: -1.15, 95% CI [-1.72, -0.59], *P* < 0.0001; Fig. 3b). The included studies showed low heterogeneity (I² = 16.4%, *P* = 0.27).

#### Adverse events (AEs)

**Fig. 4:**
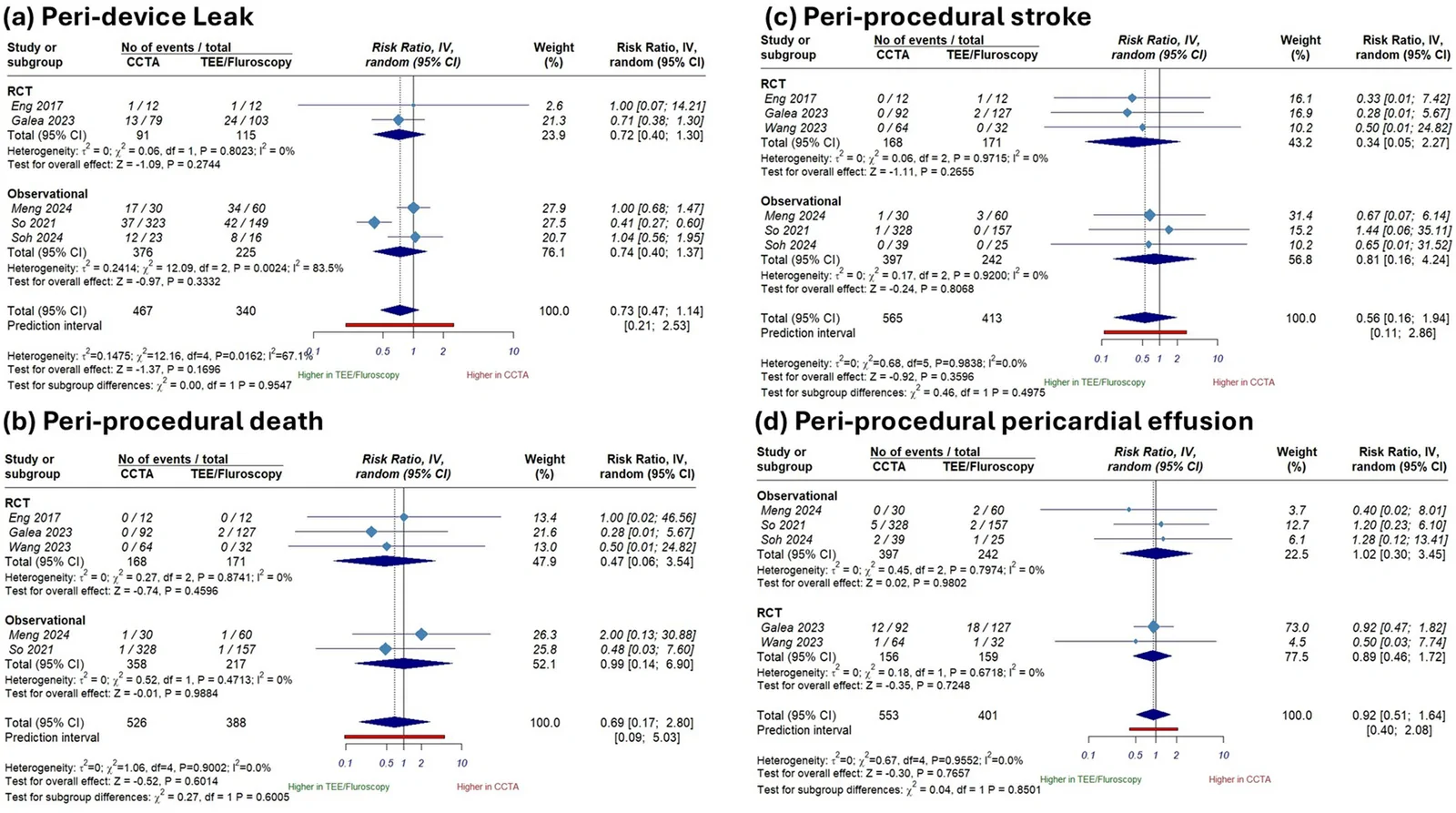
Forest plot of: Peri-device leakage (**a**), Periprocedural death (**b**), Periprocedural stroke (**c**), and Periprocedural pericardial effusion (**d**)

The pooled analysis showed no significant differences between the two groups regarding any AEs(RR: 0.65, 95% CI [0.34, 1.25], *P* = 0.19; Fig. 3c), risk of device embolization (RR: 0.56, 95% CI [0.15, 2.06], *P* = 0.38; Fig. 3d), peri-device leak (RR: 0.73, 95% CI [0.47, 1.14], *P* = 0.16; Fig. 4a), periprocedural death, (RR: 0.69, 95% CI [0.17, 2.80], *P* = 0.6; Fig. 4b), periprocedural stroke (RR: 0.56, 95% CI [0.16, 1.94], *P* = 0.35; Fig. 4c), periprocedural pericardial effusion (RR: 0.92, 95% CI [0.51, 1.64], *P* = 0.76; Fig. 4d). There was no observed heterogeneity (I^2^ = 0%) across studies in any adverse events, device embolization, periprocedural death, periprocedural stroke, or periprocedural pericardial effusion, whereas there was substantial heterogeneity (I^2^ = 67.1%) across studies in peri-device leak. The sensitivity analysis using the leave-one-out method showed robustness of the overall results in all adverse events, as no single study omission affected the direction or significance of the results (Figures S5, S6, S7, S8, S9, and S10). Subgroup analysis by study design yielded results consistent with the overall analysis for all adverse events, except that the RCT subgroup showed a statistically significantly lower rate of adverse events in the CCTA group (RR: 0.30, 95% CI [0.09, 0.99], *P* = 0.04).

#### Radiation dose, fluoroscopy time, and contrast utilization

**Fig. 5:**
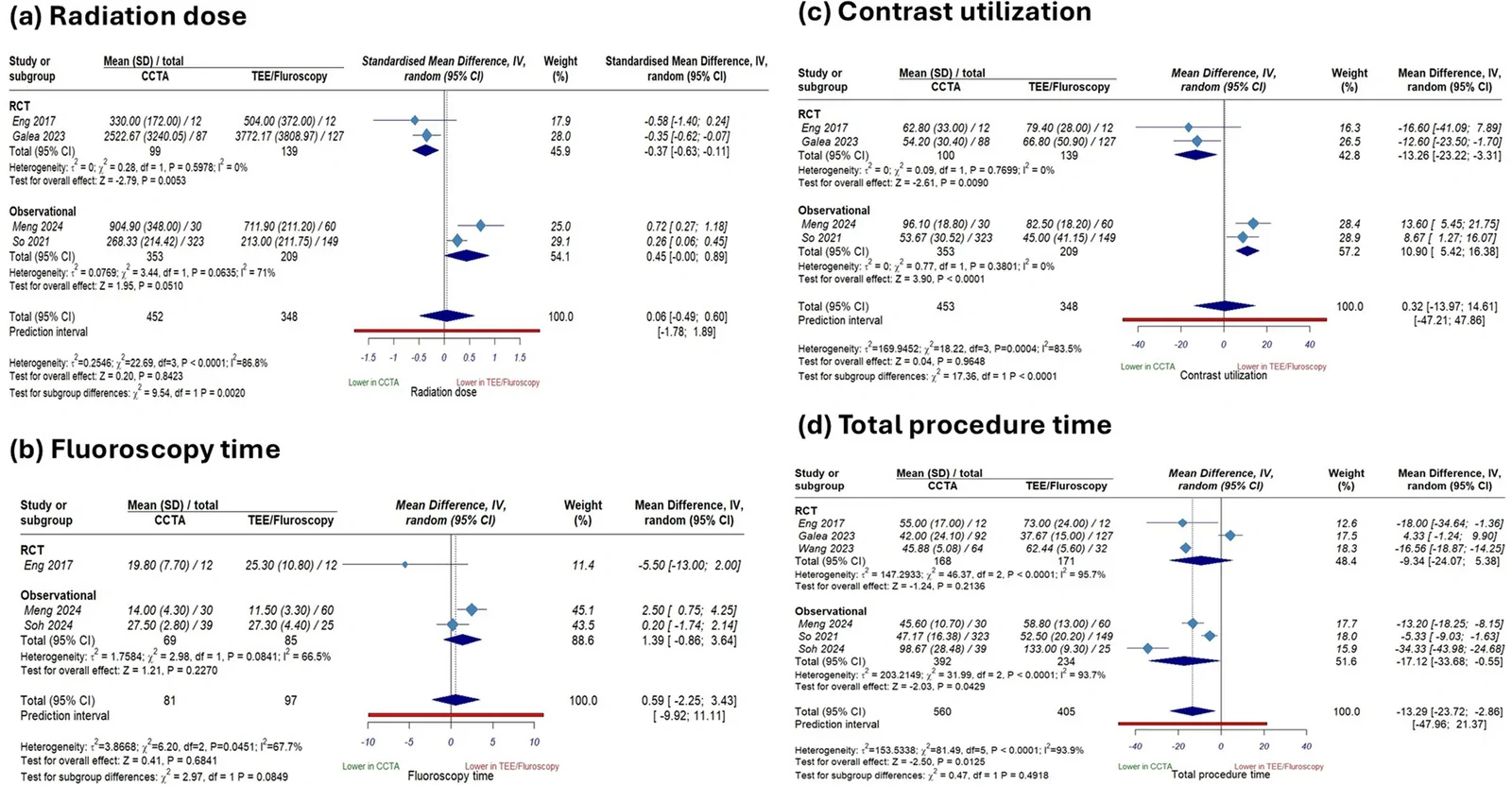
Forest plot of: Radiation dose (**a**), Fluoroscopy time (**b**), Contrast utilization (**c**), and Total procedural duration (**d**)

The pooled analysis indicated no significant differences between the two groups in radiation dose (SMD: 0.06, 95% CI [-0.49, 0.60], *P* = 0.84; Fig. 5a), fluoroscopy time (MD: 0.59, 95% CI [-2.25, 3.43], *P* = 0.68; Fig. 5b), or contrast utilization (MD: 0.32, 95% CI [-13.97, 14.61], P *=* 0.96; Fig. 5c). There was considerable heterogeneity across the included studies in radiation dose (I² = 86.8%, *P* < 0.0001) and contrast utilization (I² = 83.5%, *P* = 0.0004), whereas substantial heterogeneity was observed in fluoroscopy time (I² = 67.7%, *P* = 0.04). Sensitivity analysis using the leave-one-out method showed robustness of the overall results in all adverse events, as no single study omission affected the direction or significance of the results; yet it did not resolve the heterogeneity (Figures S11, S12, and S13). By study design, RCTs showed a significant reduction in radiation dose with CCTA (SMD − 0.37, 95% CI [-0.63, -0.11]), while observational studies showed no significant difference (SMD 0.45, 95% CI [0.00, 0.89]). Additionally, by study design, RCTs showed a significant reduction in contrast utilization with CCTA (MD -13.26, 95% CI [-23.22, -3.31]), while observational studies showed a significant increase in contrast utilization with CCTA (MD 10.90, 95% CI [5.42, 16.38]). Regarding fluoroscopy time, subgrouping by study design yielded results consistent with the overall analysis.

#### Total procedural time

CCTA was significantly associated with reduced total procedural time compared to TEE/fluoroscopy (MD: -13.29, 95% CI [-23.72, -2.86], *P* = 0.01; Fig. 5d). However, considerable heterogeneity was observed across studies (I² = 93%, *P* < 0.01). Sensitivity analysis did not resolve the heterogeneity when omitting individual studies, whereas omitting Wang et al.[17] led to a non-significant difference between the two groups (MD -12.70, 95% CI [-25.57, 0.16], *P* = 0.05; Figure S14). Subgroup analysis by study design showed that the significant difference was maintained only in the observational subgroup, not the RCT subgroup.

## Discussion

We investigated the effect of adding CCTA to the pre-procedural assessment of patients undergoing LAAO. Our systematic review and meta-analysis included six studies with 978 patients. Our analysis revealed that using CCTA-guided LAAO was associated with significantly lower rates of device resizing, procedural time, and number of intraoperative angiographic positions. Moreover, CCTA was associated with a statistically significant, albeit slight, higher rate of implantation success compared with TEE/fluoroscopy. There were no significant differences in the rates of compression, peri-device leak, or the incidence of any adverse events. Furthermore, there was no significant difference in the radiation dose, fluoroscopy time, or contrast utilization.

Currently, TEE is the standard imaging modality for occluding the left atrium, being a crucial modality for thrombus exclusion [27] underscoring the expanding role of multimodality imaging in optimizing LAAO workflows. However, other modalities, such as CCTA and CMR, have gained popularity, providing the same key data as TEE. CMR was shown to offer an alternative noninvasive imaging modality for assessing LAA thrombi compared to TEE [28]. Additionally, CMR eliminates the risk of ionizing radiation and contrast agents associated with CCTA, making it a promising modality for those with renal impairment [29]. CCTA with delayed contrast imaging was shown to perform similarly to TEE when used to rule out atrial thrombus [30, 31]. However, the time between the CCTA and the LAAO procedure is essential, as thrombi could form in the LAA if the interval is prolonged, especially in patients with atrial fibrillation.

Determination of the widest diameter of the LAA is essential for accurate sizing of the Watchman devices [27]. Wang et al. showed that CT measurements of LAA width were 2.7 ± 2.2 mm wider than TEE measurements [17]. Eng et al. also found that the difference between CT and TEE measurements was 2.2 ± 1.8 mm [15]. This measurement is affected by preload, as measured by the LAA, and undergoing TEE requires fasting. Thus, LAA is underinflated, and measurements are undersized [32]. On the other hand, patients undergoing CT maintain their usual hydration, matching the actual LAA conditions [14, 33]. All of this could explain the observed statistically significant reduction in the risk of device resizing when CCTA was added to the pre-procedural assessment of patients undergoing LAAO. Also, we found that CCTA-guided LAAO resulted in a significantly shorter procedure time. Similarly, Sattar et al. found substantially shorter procedure time in the group using CCTA for pre-procedural planning [34]. This could be explained by the lower attempts to resize the device, thus reducing the overall procedure time.

In this analysis, we found a statistically significantly higher rate of implantation success with CCTA compared with TEE/fluoroscopy. This is comparable to the findings of Clarke et al., in which preprocedural CT/CMR was independently associated with a statistically significant increased likelihood of implantation success, reflecting deployment and release of the implantation device; device success reflecting peri-device leak of less than 5 mm; and procedural success reflecting no in-hospital major adverse events compared to the nonuse of CT/CMR for LAAO [35]. Although CCTA guidance led to a slight improvement in implantation success (~ 6%), this finding should be interpreted cautiously. The result was statistically fragile, as a sensitivity analysis demonstrated that omitting the study by Galea et al. [25] attenuated the significance of this benefit. A higher likelihood of first-attempt success may reduce the need for device repositioning, exchanges, or recaptures, all of which can prolong procedure time, increase radiation and contrast exposure, and elevate the risk of procedural complications. This could make LAA occlusion challenging, especially in patients with chicken-wing shapes [36, 37]. For instance, patients with active LAA thrombus are not eligible for the procedure; a meta-analysis showed that CCTA sensitivity for detecting LAA thrombus is 96% [32, 38]. Moreover, the site of the septal puncture varies depending on LAA morphology; patients with retroverted chicken wing morphology require a more anterior puncture site than the other LAA morphologies [39]. CCTA provides high-resolution images and can accurately guide the septal puncture site [40]. Preprocedural CCTA assessment of the LAA allows for the accurate evaluation of the LAA [41], especially for those with more challenging anatomies, facilitating accurate device sizing and selection and ensuring the optimal transseptal puncture location, leading to proper positioning and stability of the implantation device within the LAA, which could all explain the observed superiority of additive preprocedural CCTA compared to standalone TEE for LAAO [13, 14, 18].

Despite the observed significant reduction in the risk for device resizing and procedure times, in addition to the substantial increase in implantation success for those undergoing additional preprocedural CCTA assessments, these gains must be balanced against the added costs and the logistical challenges, such as additional scheduling, data processing, and interpretation, with many hospitals lacking the necessary infrastructure and expertise for this integration. Significant institutional-level variability was observed in the application of pre-procedural CCTA, with its incorporation being more common among university hospitals, teaching hospitals, and larger institutions [35]. Additionally, the higher costs associated with preoperative CCTA incorporation could limit its widespread adoption, keeping in mind the lack of a standardized methodology in acquiring and interpreting these CT images [14, 42].

The addition of CCTA to the preoperative assessment of LAAO inevitably subjects patients to more radiation and contrast exposure. Although increased radiation exposure is a concern with CCTA, modern devices can help adjust the optimal radiation dose to achieve the highest image quality with the lowest radiation dose [14]. Our study found that both groups had comparable radiation doses, indicating the safety of CCTA. This is consistent with the findings of Eng et al. [15]. Furthermore, we found that CCTA was associated with fewer intraoperative angiographic positions per case. Similarly, Osmanick et al. showed that CCTA provided the best prediction of the fluoroscopic angles [43]. This can be explained as CCTA providing accurate images in multiple planes, followed by further processing and the formation of 3D models, enabling the selection of optimal fluoroscopic angles [44]. Lu et al. showed that personalized fluoroscopic angles guided by CT result in better efficiency and a significantly lower procedure time, with a mean difference of 64.3 min [45]. No significant differences were found regarding intraoperative fluoroscopy time, suggesting that while preoperative CCTA resulted in better anatomical assessment of the LAA, together with accurate detection of the implantation device size, it did not show significant effects on reducing the imaging requirements for the procedure itself, including transseptal puncture, sheath manipulation, and device deployment.

The incidence of AKI is a primary concern while using CCTA [46], considering the use of additional contrast. However, we found no significant difference in contrast utilization between the groups. Additionally, performing CCTA shortly before LAAO may result in cumulative contrast exposure, as the patient may require separate injections, one for the CCTA scan and the other for the procedure itself; thus, the timing of CCTA is essential, especially for those with renal impairment.

Although contrast use was similar between CCTA-guided and non-guided groups, concerns about contrast-induced AKI (CI-AKI) remain, especially in patients with reduced kidney function. Most studies excluded participants with advanced chronic kidney disease, limiting the extent to which we can apply these findings. Renal outcomes, such as changes in serum creatinine or CI-AKI rates, were also rarely reported, making it hard to draw firm safety conclusions. While strategies like checking renal function and ensuring adequate hydration help reduce risk, the timing of CCTA remains essential, especially if patients receive separate contrast doses for imaging and the procedure. Future research should include patients with a broader range of kidney function and consistently report nephrotoxicity outcomes to better understand the renal safety of CCTA in real-world LAAO practice.

Achieving sufficient compression is important, as compression below 10% is associated with an increased incidence of peri-device leaks [47]. Our study found no significant difference in compression ratios or in the rate of peri-device leaks. Conversely, Croix et al. found significantly lower rates of peri-device leaks in the CCTA group [48]. However, the definition of peri-device leak and its consequences requires further investigation, given the variation in definitions [49]. The peri-device leak is associated with the incidence of ischemic events. However, since patients with peri-device leakage are placed on anticoagulant therapy, the number of ischemic events is relatively low [47].

Accurate placement of occluding devices is essential, as deep placement is associated with an increased risk of device-related thrombus [50]. Although this is not a common complication of LAAO, it is associated with a fivefold increase in the risk of ischemic adverse events [51]. Furthermore, inaccurate placement could result in device embolization, a potentially life-threatening complication [52]. We found that the safety outcomes were comparable between both groups; there was no significant difference in the incidence of adverse events, stroke risk, embolization, or mortality. Furthermore, CCTA is a non-invasive procedure. It could minimize patient discomfort, anesthesia, and the risk of aspiration or esophageal perforation associated with TEE [53]. It has recently been shown that CT is superior to TEE in the post-procedural assessment of patients following LAA occlusion [54]. Furthermore, experts recently provided recommendations for the use of CCTA in the pre-procedural evaluation of LAAO. This can guide physicians aiming to use CT in the pre-procedural assessment of LAAO [14, 25].

While CCTA has demonstrated potential benefits in procedural planning, such as reducing device resizing, minimizing implantation attempts, and shortening overall procedure time, its routine use raises important economic and logistical considerations that merit further scrutiny. The implementation of CCTA requires not only advanced imaging infrastructure but also specialized software for 3D reconstruction and staff trained in image post-processing and interpretation. These resource demands may pose barriers in low-volume or resource-limited centers [55]. Moreover, concerns about additional radiation and contrast exposure must be balanced against potential downstream reductions in procedural time and complications. Recent studies suggest that CCTA-guided planning may yield a net procedural efficiency benefit, particularly in anatomically complex cases. This is particularly true during early operator learning curves, when more accurate preplanning can mitigate uncertainty and reduce intra-procedural adjustments, and in high-volume centres, where cumulative time savings improve procedural throughput [56].

### Strengths

Our study has several strengths. It provides a comprehensive synthesis of randomized controlled trials and observational studies, thereby enhancing its applicability across research and clinical settings. We strictly adhered to PRISMA guidelines and registered our protocol in PROSPERO, ensuring transparency and methodological rigor. Most of the studies included were of good quality, and we conducted multiple sensitivity analyses to confirm the robustness of our findings. Furthermore, we performed subgroup analyses based on study design (RCT vs. observational) to explore sources of heterogeneity. By evaluating a wide range of procedural and safety outcomes, the study offers a balanced assessment of CCTA-guided LAAO.

### Limitations

Our study has some limitations. First, there was substantial heterogeneity in the analysis of key outcomes, such as procedural time and radiation dose, which could compromise the reliability of the results. Second, all included studies used a watchman device, except for Galea et al. [25], who used both the Amplatzer Amulet and the Watchman device, and Soh et al. [26], who exclusively used the Amplatzer Amulet device. Thus, our findings may not be fully applicable to procedures involving other LAAO devices. Importantly, the Amplatzer Amulet and other occluders differ from the WATCHMAN in their design, sizing protocols, anchoring mechanisms, and deployment strategies. These procedural and anatomical differences could influence the utility and interpretation of preprocedural imaging. Unfortunately, the available data were insufficient to support a device-specific subgroup analysis, and therefore, caution is warranted when generalizing our results beyond the WATCHMAN platform. Also, most of the studies did not include patients with renal disorders. Thus, this may not apply to patients with chronic kidney failure. Additionally, heterogeneity in control group imaging modalities across studies, ranging from TEE alone to TEE plus fluoroscopy, may have influenced the pooled estimates; however, this could not be evaluated further because only a single study used TEE alone, yielding insufficient data for a reliable subgroup analysis.

Additionally, despite pooling data on contrast utilization across groups, limited evidence was available on contrast-related renal risks, a significant concern requiring future investigation and comparison with other non-contrast-requiring imaging modalities, such as CMR. Some of the studies used 3D models to visualize the LAA. This helps improve the learning curve and acquire accurate LAA diameters [13]. However, the applicability of such an approach might be limited in centers with limited resources or without highly qualified staff. Additionally, most included studies reported follow-up data limited to 45–90 days. Data on long-term clinical outcomes (beyond 90 days), particularly regarding device-related thrombus (DRT), late ischemic stroke, systemic embolism, and the durability of LAA sealing, were unavailable. Additionally, while the rates of peri-device leak appeared similar between groups, these findings were based on short-term imaging using heterogeneous definitions and modalities. Consequently, whether CCTA-guided planning reduces device-related thrombus (DRT) or late thromboembolic events remains hypothetical and cannot be determined from the available data. Importantly, sensitivity analysis demonstrated that the observed statistical significance for implantation success was attenuated when the Galea et al. study was excluded, suggesting that the result is not robust and should be interpreted with caution.

Future studies aim to assess whether CCTA-guided LAAO planning has a measurable impact on long-term outcomes, including device-related thrombus, ischemic stroke, and persistent peri-device leak. Trials with extended follow-up, standardized imaging definitions, and consistent reporting of clinical endpoints are needed to validate the hypothesized benefits of improved preprocedural planning. In addition, evaluating the role of CCTA across different occluder devices and patient populations, as well as its cost-effectiveness in diverse healthcare settings, will help define its optimal use in clinical practice.

## Conclusion

Our study showed that CCTA-guided LAAO was associated with significantly, albeit slightly, higher implantation success rates, along with considerably lower rates of device resizing, procedural time, and intraoperative angiographic positions. Furthermore, there were no significant differences in radiation dose, contrast utilization, compression, peri-device leak, or the incidence of any adverse events, supporting its potential safety. The limited follow-up (≤ 90 days) restricted conclusions on long-term outcomes. Future long-term randomized controlled trials are needed to assess overall cost-effectiveness, as well as the additional logistical, contrast, and radiation burden, to better inform clinical decision-making and the potential for widespread adoption.

## Supplementary Information

Below is the link to the electronic supplementary material.

Supplementary Material 1 (DOCX 1.45 MB)

## Data Availability

The data is available upon reasonable request from the corresponding author.
