# Supplementary material for: The additive value of preprocedural cardiac computed tomography angiography (CCTA) planning in percutaneous left atrial appendage occlusion: a systematic review and meta-analysis
Source: J Interv Card Electrophysiol. 2026 Mar 28;69(7):2071–84. doi: 10.1007/s10840-026-02273-2 (PMC13687204; doi:10.1007/s10840-026-02273-2)
Supplement: Supplementary file 1 [file 10840_2026_2273_MOESM1_ESM.docx]

**Title.**

**The Additive Value of Preprocedural Cardiac Computed Tomography (CCT) Planning in Percutaneous Left Atrial Appendage Occlusion: A Systematic Review and Meta-Analysis**

**Running Title.**

Additive Value of CCTA in LAAO *Table S1. Search Strategy*

| Database | Search Terms | Number pf Results |
| --- | --- | --- |
| PubMed | ("computed tomography" OR CT OR CCT OR CCTA) AND ("left atrial appendage occlu*" OR "left atrial appendage clos*" OR "LAA occlu*" OR "LAA clos*" OR LAAO OR LAAC OR watchman OR atriclip OR plaato OR lariat OR amulet OR "cardiac plug" OR ultraseal) AND ("transesophageal echo*" OR TEE OR "echocardiography-guided" OR ultrasound OR "ultrasound-guided" OR "echo*") | 378 |
| Cochrane Library | ("computed tomography" OR CT OR CCT OR CCTA) AND ("left atrial appendage occlu*" OR "left atrial appendage clos*" OR "LAA occlu*" OR "LAA clos*" OR LAAO OR LAAC OR watchman OR atriclip OR plaato OR lariat OR amulet OR "cardiac plug" OR ultraseal) AND ("transesophageal echo*" OR TEE OR "echocardiography-guided" OR ultrasound OR "ultrasound-guided" OR "echo*") | 13 |
| Scopus | ("computed tomography" OR CT OR CCT OR CCTA) AND ("left atrial appendage occlu*" OR "left atrial appendage clos*" OR "LAA occlu*" OR "LAA clos*" OR LAAO OR LAAC OR watchman OR atriclip OR plaato OR lariat OR amulet OR "cardiac plug" OR ultraseal) AND ("transesophageal echo*" OR TEE OR "echocardiography-guided" OR ultrasound OR "ultrasound-guided" OR "echo*") | 423 |
| Web of Science | ("computed tomography" OR CT OR CCT OR CCTA) AND ("left atrial appendage occlu*" OR "left atrial appendage clos*" OR "LAA occlu*" OR "LAA clos*" OR LAAO OR LAAC OR watchman OR atriclip OR plaato OR lariat OR amulet OR "cardiac plug" OR ultraseal) AND ("transesophageal echo*" OR TEE OR "echocardiography-guided" OR ultrasound OR "ultrasound-guided" OR "echo*") | 257 |
| Embase | ("computed tomography" OR CT OR CCT OR CCTA) AND ("left atrial appendage occlu*" OR "left atrial appendage clos*" OR "LAA occlu*" OR "LAA clos*" OR LAAO OR LAAC OR watchman OR atriclip OR plaato OR lariat OR amulet OR "cardiac plug" OR ultraseal) AND ("transesophageal echo*" OR TEE OR "echocardiography-guided" OR ultrasound OR "ultrasound-guided" OR "echo*") | 543 |
| Total |  | 1614 |

*Table S2. The ROBINS-I assessment for observational studies.*

| Domain | Meng 2024 | Soh 2024 | So 2021 |
| --- | --- | --- | --- |
| Confounding | Moderate | High | Serious |
| Selection of Participants | Moderate | Low | Low |
| Classification of Interventions | Low | Low | Low |
| Deviations from Intended Interventions | Low | Low | Low |
| Missing Data | Low | Moderate | Low |
| Measurement of Outcomes | Low | Low | Low |
| Selection of Reported Results | Moderate | Low | Low |
| Overall Risk of Bias | Moderate | High | Serious |

*Figure S1. RoB.2 assessment for RCTs*

*Figure S2. Leave-one-out sensitivity analysis of implantation success*

Figure S3. *Leave-one-out sensitivity analysis of device resizing.*

Figure S4. Leave-one-out sensitivity analysis of compression.

*Figure S5. Leave-one-out sensitivity analysis of any adverse events.*

Figure S6. Leave-one-out sensitivity analysis of devise embolization.

Figure S7. Leave-one-out sensitivity analysis of devise embolization.

Figure S8. Leave-one-out sensitivity analysis of periprocedural death.

Figure S9. Leave-one-out sensitivity analysis of periprocedural stroke.

Figure S10. Leave-one-out sensitivity analysis of periprocedural pericardial effusion.

**Figure S11.** Leave-one-out sensitivity analysis of radiation dose.

*Figure S12**.* Leave-one-out sensitivity analysis of *fluoroscopy time*.

Figure S13. Leave-one-out sensitivity analysis of contrast utilization.

*Figure S14.*  Leave-one-out sensitivity analysis of total procedure time.
